# RNA Sequencing-Based Bulked Segregant Analysis Facilitates Efficient D-genome Marker Development for a Specific Chromosomal Region of Synthetic Hexaploid Wheat

**DOI:** 10.3390/ijms19123749

**Published:** 2018-11-26

**Authors:** Ryo Nishijima, Kentaro Yoshida, Kohei Sakaguchi, Shin-ichi Yoshimura, Kazuhiro Sato, Shigeo Takumi

**Affiliations:** 1 Graduate School of Agricultural Science, Kobe University, Rokkodai 1-1, Nada, Kobe 657-8501, Japan; ryosnishijima@gmail.com (R.N.); k.sakaguchi@brown.plala.or.jp (K.S.); shin.82353@gmail.com (S.-i.Y.); 2 Institute of Plant Science and Resources, Okayama University, Kurashiki, Okayama 710-0046, Japan; kazsato@rib.okayama-u.ac.jp

**Keywords:** allohexaploid, homoeolog, hybrid necrosis, molecular marker, wheat

## Abstract

Common wheat originated from interspecific hybridization between cultivated tetraploid wheat and its wild diploid relative *Aegilops tauschii* followed by amphidiploidization. This evolutionary process can be reproduced artificially, resulting in synthetic hexaploid wheat lines. Here we performed RNA sequencing (RNA-seq)-based bulked segregant analysis (BSA) using a bi-parental mapping population of two synthetic hexaploid wheat lines that shared identical A and B genomes but included with D-genomes of distinct origins. This analysis permitted identification of D-genome-specific polymorphisms around the *Net2* gene, a causative locus to hybrid necrosis. The resulting single nucleotide polymorphisms (SNPs) were classified into homoeologous polymorphisms and D-genome allelic variations, based on the RNA-seq results of a parental tetraploid and two *Ae. tauschii* accessions. The difference in allele frequency at the D-genome-specific SNP sites between the contrasting bulks (ΔSNP-index) was higher on the target chromosome than on the other chromosomes. Several SNPs with the highest ΔSNP-indices were converted into molecular markers and assigned to the *Net2* chromosomal region. These results indicated that RNA-seq-based BSA can be applied efficiently to a synthetic hexaploid wheat population to permit molecular marker development in a specific chromosomal region of the D genome.

## 1. Introduction

Wild relatives of common wheat (*Triticum aestivum* L.), including *Aegilops* species, constitute important genetic resources for common wheat breeding. Notably, *Aegilops tauschii* Coss. is the D-genome progenitor of common wheat, and a limited number of *Ae. tauschii* strains appear to have contributed to the speciation of common wheat through interspecific crossing to the cultivated tetraploid wheat (*Triticum turgidum* L. ssp. *durum*) strains carrying the A and B genomes and subsequent amphidiploidization about 8000 years ago in the Fertile Crescent [1,2]. Artificial replication of this evolutionary process can be achieved by generation of synthetic hexaploid wheat derived from the interspecific crosses between cultivated tetraploid wheat and *Ae. tauschii* [3]. Thus, agriculturally useful alleles of *Ae. tauschii* are directly available for common wheat breeding via the transmission from synthetic hexaploid wheat to common wheat [4,5,6,7,8,9,10]. In common wheat, the D genome is known to harbor much lower genetic diversity than do the A and B genomes, whereas introgression of the *Ae. tauschii* natural variation via synthetic hexaploid wheat has been used successfully to enlarge D-genome diversity [11,12,13,14].

*Ae. tauschii* is widely distributed, and is present from northern Syria and southeastern Turkey to western China [15,16]. Recent studies on the population structure of *Ae. tauschii* showed that only one (TauL1) of three major lineages has contributed to the wide species range [2,17,18]. The other two major lineages, TauL2 and TauL3, are restricted to the Transcaucasus/Middle East region. TauL2 includes both subspecies *tauschii* and subspecies *strangulata*, and the TauL3 accessions are limited in Georgia [18]. The *Ae. tauschii* strains associated with the origin of common wheat are assumed to be the TauL2 lineage [2,17], and only limited reproductive barriers are thought to exist between tetraploid wheat and many of the TauL2 accessions [19]. TauL1 accessions frequently carry a hybrid incompatibility gene, designated *Net2*, that triggers low-temperature-induced necrotic cell death upon interspecific hybridization with tetraploid wheat, impeding the generation of synthetic hexaploid wheat [20,21]. Thus, *Net2* is a major reproductive barrier for breeding use of the TauL1 accessions, and the development of markers closely linked to *Net2* is needed for efficient use of the TauL1 gene pool. A fine map for the *Net2* chromosomal region already has been constructed on the short arm of chromosome 2D [22].

Genomic approaches using next-generation sequencing (NGS) techniques have been applied to analysis of the genomes of the wild relatives of domesticated crops, expanding the genetic resources available for crop improvement [23]. In common wheat, the D-genome markers remain much less developed than those of the A and B genomes, whereas recent progress using the NGS technique has facilitated an increase in the number of D-genome markers [24]. RNA sequencing (RNA-seq) of the *Ae. tauschii* accessions has generated a huge number of genome-wide polymorphisms, including single nucleotide polymorphisms (SNPs) and insertions/deletions (indels); the genome-wide SNPs and indels can be efficiently anchored to the chromosomes of barley and *Ae. tauschii* [25,26,27]. The SNP- and indel-based markers are available for construction of linkage maps in the target chromosomal regions of not only *Ae. tauschii* but also the D genome of hexaploid wheat including synthetic wheat [26,27].

Bulked segregant analysis (BSA) combined with NGS allows efficient development of molecular markers linked to a genomic region associated with the target phenotype in cereals [28,29,30,31]. In maize, for example, an RNA-seq-based BSA approach has been used to construct high-density linkage maps and to screen among candidate genes for a target locus [32,33,34]. Hexaploid wheat has three closely related genomes (designated A, B, and D), each of which carries a set of highly similar genes (homoeology). Due to the genome complexity via allopolyploidy and the large proportion of repetitive DNA in polyploid wheat, whole-genome resequencing is still unviable and reduced-representation methods of NGS data have been employed in this species [35]. Recently, RNA-seq-based BSA was employed successfully for the development of molecular markers closely linked to target chromosomal genes such as a grain protein content gene (*GPC-B1*), a yellow rust resistance gene (*Yr15*), and a powdery mildew resistance gene (*Pm4b*) in tetraploid and common wheat [36,37,38]. However, a limited amount of information has been reported for the RNA-seq-based BSA approach in polyploid wheat. Here, we employed the RNA-seq-based BSA method to develop a molecular marker closely linked to *Net2*. This process used a mapping population generated from a cross of two synthetic hexaploid wheat lines that shared identical A and B genomes but contained diverse D genomes originating from two distinct pollen parents.

## 2. Results and Discussion

**Figure 1:**
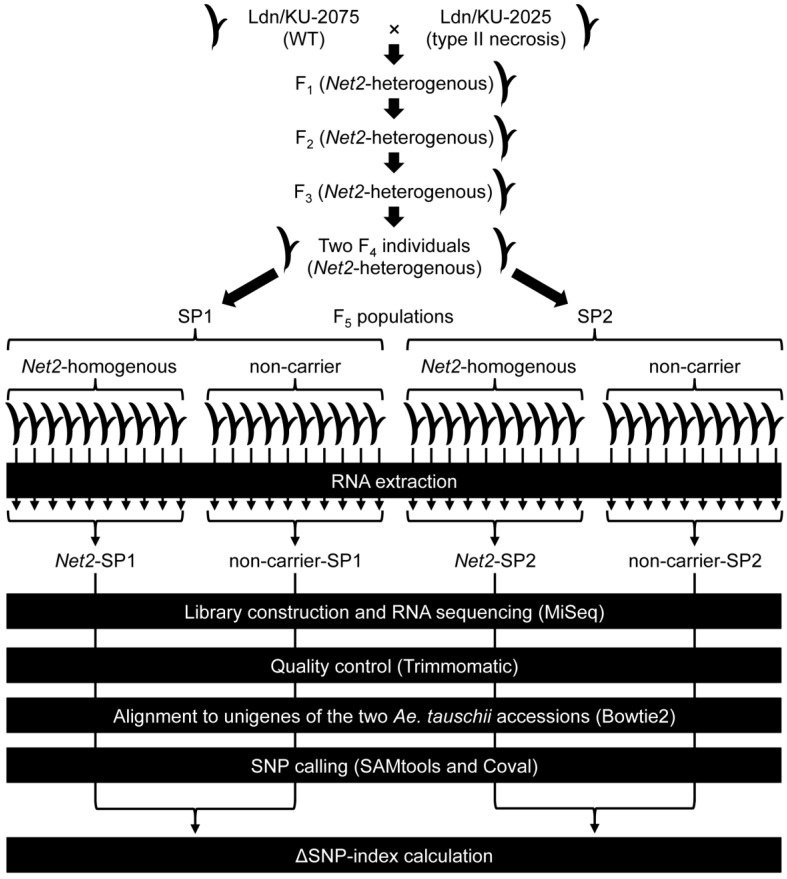
Workflow of the RNA sequencing analysis in this study.

Two synthetic hexaploid wheat lines were derived from interspecies crosses of tetraploid wheat cultivar Langdon (Ldn) and two *Ae. tauschii* accessions (KU-2075 and KU-2025). Ldn/KU-2075 and Ldn/KU-2025, respectively, showed normal phenotype (wild-type) and type-II necrosis phenotype [20]. To obtain novel molecular markers tightly linked to *Net2*, RNA sequencing of four bulks of synthetic hexaploid wheat was performed (Figure 1). Each bulk was composed of ten *Net2*-homozygous individuals or non-carriers (*net2*-homozygous) selected from two F_5_ populations between Ldn/KU-2075 and Ldn/KU-2025 (hereafter referred to as Segregating Populations SP1 and SP2). The two bulks of each SP (*Net2*-SP1, non-carrier-SP1, *Net2*-SP2, and non-carrier-SP2) were sequenced twice; the bulks were designated as follows: *Net2*-SP1-1st, *Net2*-SP1-2nd, non-carrier- SP1-1st, non-carrier-SP1-2nd, *Net2*-SP2-1st, *Net2*-SP2-2nd, non-carrier-SP2-1st, and non-carrier-SP2-2nd (Table 1). In each experiment, 4.06 million to 5.22 million paired-end reads were generated. After quality filtering, 2.80 million to 3.71 million high-quality reads were acquired. These reads were aligned with transcripts of the two parental synthetic hexaploid *Ae. tauschii* accessions, KU-2075 and KU-2025, transcriptomes that had been de novo assembled in our previous study [23]. In each experiment, 2.02 million to 2.76 million and 1.93 million to 2.68 million reads were aligned to the KU-2075 and KU-2025 transcriptomes, respectively. The alignment output files of the first and second runs were merged for each alignment pair of bulk and transcript. On average, 292,678 and 278,690 SNPs were detected on KU-2075 and KU-2025 transcripts, respectively (Table 2). Of these, 290,712 (99.33%) and 276,700 (99.29%) SNPs on the KU-2075 and KU-2025 transcripts (respectively) were anchored to the *Ae. tauschii* genome. SNP sites in the eight alignment pairs of bulk and transcript (e.g., *Net2*-SP1 vs. KU-2075), as shown in Table 3, were integrated with the genome sequence, yielding a total of 319,808 non-redundant (NR) SNP sites on the seven chromosomes (Table 3). This section may be divided by subheadings. It should provide a concise and precise description of the experimental results, their interpretation as well as the experimental conclusions that can be drawn.

To distinguish D-genome-specific variations from homoeologous polymorphisms between the tetraploid AB and diploid D genomes, the RNA-seq raw read data of Ldn, the tetraploid parental accession of the synthetic wheat lines, were processed as described above. Out of the total of 6.32 million read pairs obtained, 4.37 million read pairs passed the quality filtering (Table 1). To KU-2075 and KU-2025 transcripts, 2.97 million and 2.66 million reads were aligned, respectively.

Out of the 429,346 and 350,871 SNPs identified on KU-2075 and KU-2025 transcripts (respectively), 421,957 (98.28%) and 345,657 SNPs (98.51%) (respectively) were anchored to the *Ae. tauschii* genome (Table 2). Based on these SNPs derived from tetraploid wheat reads, and on the SNPs between the two *Ae. tauschii* accessions reported in our previous study [27], NR SNPs identified in synthetic hexaploid bulks were classified into homoeologous polymorphisms and D-genome-specific allelic variation. Out of the 319,808 SNPs, 17,927 were assigned to the allelic variation on the D genome, and 211,930 were grouped into the homoeologous polymorphisms, while the remained 89,951 did not fall into either of these two classes (“unclassified”; Table 3).

**Figure 2:**
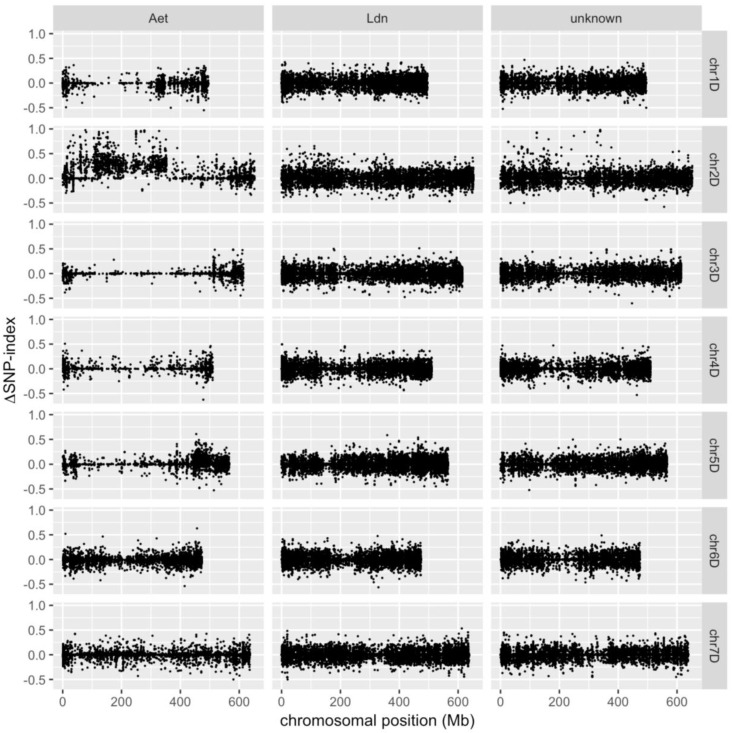
Distribution of ΔSNP-index values along the *Ae. tauschii* chromosomes. The three categories of SNPs (D-genome-specific allelic variations, homoeologous polymorphisms, and unclassified SNPs) are designated as “Aet”, “Ldn”, and “Unknown”, respectively.

**Figure 3:**
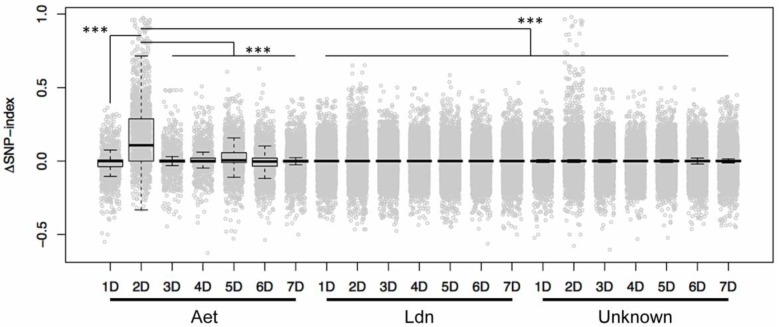
Box and dot plots for ΔSNP-index values based on the seven chromosomes and on the SNP classifications in Figure 2. D-genome-specific SNPs on chromosome 2D had significantly higher ΔSNP-index values than SNPs on the other six chromosomes, homoeologous SNPs, and unclassified SNPs. *** *p* < 0.001 by the Steel-Dwass test.

SNP-index values were computed for each of the eight alignment pairs, and then ΔSNP-index values were calculated for the comparisons between *Net2*-homogenous bulks and non-carrier bulks, yielding indices for *Net2*-SP1 minus non-carrier-SP1 and *Net2-*SP2 minus non-carrier-SP2. The average of the four ΔSNP-index values at each SNP site was mapped to the *Ae. tauschii* genome (Figure 2). The ΔSNP-index distribution on the chromosomes indicated that SNPs with high ΔSNP-index values tended to be located on the short arm of chromosome 2D, where the *Net2* locus resides (Figure 2). Comparison of ΔSNP-index values among D-genome-specific SNPs showed that the D-genome-specific SNPs on chromosome 2D possessed significantly higher (Steel-Dwass test, *p* < 0.001) ΔSNP-index values than those on the other six chromosomes (Figure 3). Theoretically, homozygous chromosomal regions account for 87.5% of the total genome in an F_4_ individual derived from two parents. D-genome-specific SNP sites with low ΔSNP-index values appeared to already have been fixed as homozygous for the KU-2075 or KU-2025 allele by the F_4_ generation of the synthetic hexaploid populations. The ΔSNP-index values of D-genome-specific SNPs on chromosome 2D also were significantly higher (Steel-Dwass test, *p* < 0.001) than those of homoeologous and unclassified SNPs (Figure 3). Moreover, most of the homoeologous and unclassified polymorphisms showed ΔSNP-index values of approximately zero (Figure 3), indicating that homoeologous polymorphisms had been efficiently removed from candidate SNPs for development of molecular markers, since such candidate SNPs should have possessed high ΔSNP-index values. Low ΔSNP-index values at homoeologous polymorphic sites were likely due to the similar allele frequency of A- and B-genome-derived reads between the *Net2*-homogenous and non-carrier bulks, which thereby offset the SNP-index values of each other.

**Figure 4:**
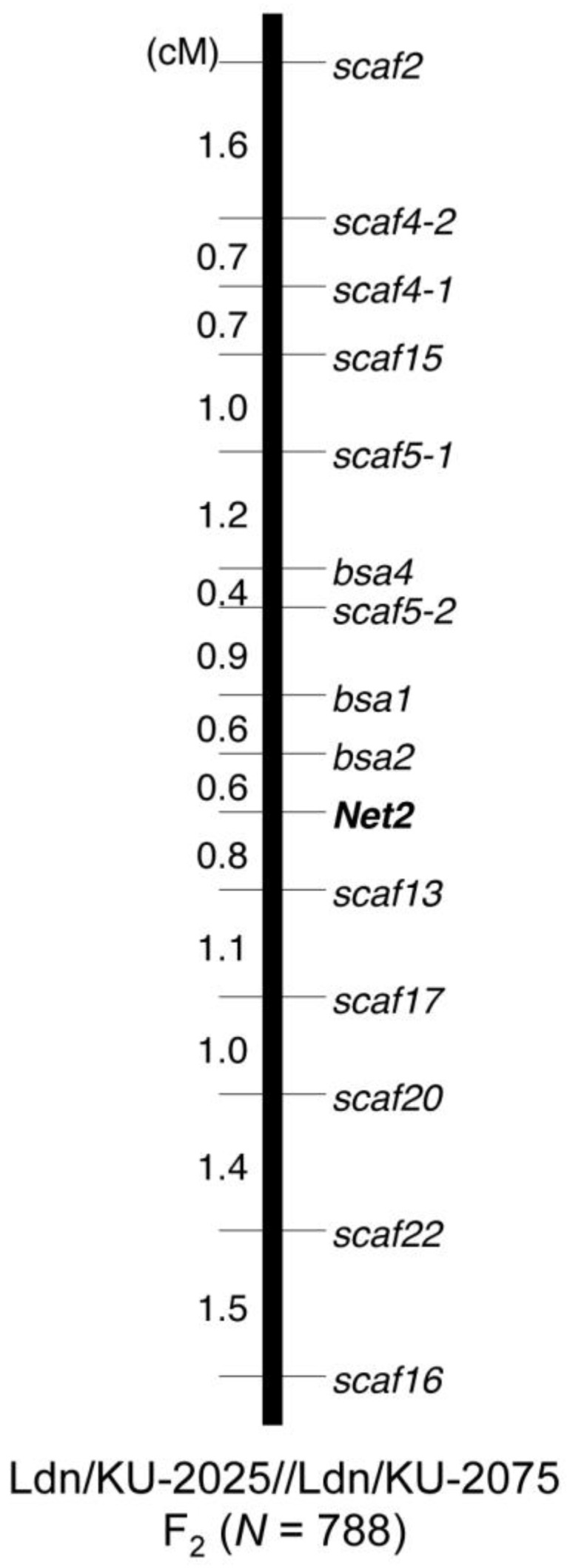
Genetic map of the short arm of chromosome 2D in the Ldn/KU-2025//Ldn/KU-2075 population. The three newly developed markers are designated with “*bsa*” prefixes.

To assess whether SNPs with high ΔSNP-index values can be used as molecular markers for the *Net2* locus, derived cleaved amplified polymorphic sequence (dCAPS) markers were designed from these SNPs. Within the physical region from 81.8 Mb to 83.3 Mb on chromosome 2D that was defined by the two markers noted in our previous study [22], 208 SNPs were detected in the present study, including 10 D-genome specific, 144 homoeologous, and 54 unclassified SNPs. The ΔSNP-index values of this subset of SNPs ranged from −0.4025 to 0.5425. Four of the D-genome-specific SNPs with ΔSNP-index values higher than 0.38 were converted into dCAPS markers (Table 4), and three of these markers (designated *bsa1*, *bsa2*, and *bsa4*) were successfully genotyped and mapped in the Ldn/KU-2025//Ldn/KU-2075 population (Figure 4). All three of these markers were anchored within 2.5 cM distal to the *Net2* locus, and two of these markers were closer to *Net2* than to *scaf52*, the most closely linked markers in our previous study [22]. This result indicates that SNPs derived from RNA-seq-based BSA of synthetic hexaploid wheat can be used for molecular marker development in a specific chromosomal region of the D genome.

The distribution of ΔSNP-index values was not continuous (Figure 2), even in a single transcript. The discontinuous pattern was due to the homoeologous and nonpolymorphic RNA-seq reads, which could be derived from homoeologous regions in the A and B genomes and overlap with the reads containing the D-genome-specific SNPs. For complete masking of the overlapping reads from the A and B genomes, precise alignment to all three genomes of hexaploid wheat would be needed. In the present study, although transcripts of the parental diploid species were used as the reference sequences, our strategy succeeded in efficiently detecting candidate SNPs on a specific chromosomal region of synthetic hexaploid wheat. Therefore, this strategy of RNA-seq-based BSA is expected to be applicable to other synthetic polyploids derived from crosses among tetraploid and/or diploid wheat relatives.

Taken together, these data demonstrate that RNA-seq-based BSA can be applied to synthetic hexaploid wheat for the development of molecular markers in specific chromosomal regions. The use of a single tetraploid wheat cultivar as a parental line for synthetic hexaploid wheat effectively cancelled the increase in ΔSNP-index values at homoeologous polymorphic sites. To date, a large number of synthetic wheat hexaploids have been produced as part of efforts to enlarge the D-genome genetic diversity [5,6,40]. Epistatic interactions can occur between the parental genomes in the newly synthesized allopolyploids [41], and several phenotypes specific to the synthetic wheat hexaploids have been reported [20,42]. RNA-seq and subsequent de novo transcriptome assembly can be performed even in wild diploid wheat relatives with no reference sequences. This strategy would facilitate molecular marker development in diverse wheat synthetics with various genome constitutions. Thus, RNA-seq-based BSA is expected to serve as a rapid and efficient approach for genetic evaluation of target traits from wild wheat relatives with allopolyploid backgrounds.

## 3. Materials and Methods

### 3.1. Plant Materials

Two synthetic hexaploid wheat lines derived from interspecies crosses of tetraploid wheat cultivar Langdon (Ldn) and two *Ae. tauschii* accessions (KU-2075 and KU-2025) were used in this study. These lines were produced in our previous study [21]. A synthetic hexaploid line (Ldn/KU-2075) showed normal growth, whereas another synthetic hexaploid line (Ldn/KU-2025) exhibited the type-II necrosis phenotype when grown at low temperature [21]. An F_2_ mapping population was generated through a cross of the two synthetic hexaploid lines [22]. Subsequently, the F_3_ and F_4_ generations were developed from selfing F_2_ and F_3_ individuals (respectively) heterozygous for the chromosomal region of *Net2*, a type-II necrosis-causing gene [22]. Two F_5_ populations (SP1 and SP2) were generated through selfing of two independent F_4_ individuals heterozygous for the *Net2* region (Figure 1).

### 3.2. Library Construction and RNA Sequencing

Ten *Net2*-homozygous individuals and ten non-carriers (*net2*-homozygous) each were selected from the SP1 and SP2 populations. Total RNA was extracted from leaves of each of the forty selected plants at the seedling stage using a Plant Total RNA Extraction Miniprep System (Viogene, Taipei Hsien, Taiwan, ROC). The resulting RNA preparations were pooled independently into four bulk preparations according to the genotype of the *Net2* region and to the SP source population (*Net2*-SP1, non-carrier-SP1, *Net2*-SP2, and non-carrier-SP2). A total of 8 μg of each bulk preparation of RNA was used to construct paired-end libraries using TruSeq RNA Library Preparation Kit v2 (Illumina, San Diego, CA, USA). The libraries were sequenced twice for 300 cycles × 2 on an Illumina MiSeq sequencer with 300-bp paired-end reads. The read data was deposited to the DDBJ Sequence Read Archive under the accession number DRA007501. RNA sequencing data of Ldn, which was obtained in our previous report [43] and deposited under the accession number DRA007097, also was used for the subsequent analysis.

### 3.3. Alignment of RNA-seq Reads to de Novo Assembled Transcripts of the Parental Ae. tauschii Accessions

Low-quality bases (average Phred quality score per 4 bp < 30), adapter sequences, and reads < 100 bp were removed from the short reads using the Trimmomatic version 0.32 tool [44]. RNA-seq analysis of KU-2075 and KU-2025 for de novo transcriptome assembly was described in our previous study [27], and the corresponding sequencing data was obtained from the DDBJ Sequence Read Archive DRA004604. The quality-filtered reads of each bulk and Ldn were aligned to the transcripts of KU-2075 and KU-2025 using the Bowtie2 tool [45] with local alignment, obtaining bam outputs.

### 3.4. Identification of D-genome Specific SNPs and Calculation of ΔSNP-Index

After merging the two bam files of each bulk library using SAMtools software (version 1.9; La Jolla, CA, USA) with the command “samtools merge”, SNPs were called from the alignment files using SAMtools and Coval software (version 1.5; Iwate, Japan) with option “-freq 0.1 -m 1000000 -n 10” [46,47]. The bam files of the synthetic hexaploid bulks were supposed to hold homoeologous polymorphisms and allelic variations on the D genome, whereas the alignment outputs of Ldn would contain homoeologous polymorphisms only. We assumed that, of the SNPs derived from the bulks, SNPs also detected in the pairwise comparison of KU-2075 and KU-2025 would represent D-genome specific polymorphisms, and SNPs also found in the alignment files of Ldn would represent homoeologous polymorphisms. The transcript sequences of the two *Ae. tauschii* accessions were mapped to the reference genome of *Ae. tauschii* [39] using GMAP version 2013-03-31 software (South San Francisco, CA, USA) [48,49], and the identified SNPs were anchored to the *Ae. tauschii* genome based on the GMAP outputs. The allele frequency at the SNP sites, a value designated as the SNP-index [28,29], was calculated in each of the eight respective alignment pairs, and the difference in the SNP-index values of the contrasting bulks (*Net2*-homogenous *minus* non-carrier bulk alignment, e.g., *Net2*-SP1-KU-2075 *minus* non-carrier-SP1-KU-2075) was defined as the ΔSNP-index by analogy to the work of Takagi et al. [29]. RStudio ver. 0.99.902 [50] with R software ver. 3.3.1 [51] was used for statistical analyses and ΔSNP-index plotting of the *Ae. tauschii* chromosomes.

### 3.5. Molecular Marker Development and Genotyping

In our previous study [22], the *Net2* locus was fine-mapped within a 0.6-cM region on the short arm of chromosome 2D. The two most closely linked markers were assigned to the *Ae. tauschii* genome [39] using the BLASTN search function of the BLAST+ software (Bethesda, MD, USA) [52] to define the physical region where the *Net2* locus was assumed to reside. D-genome-specific SNPs of high ΔSNP-index values within this chromosomal region were converted into derived cleaved amplified polymorphic sequence (dCAPS) markers (Table 4), and these novel markers were used to genotype 788 individuals of the Ldn/KU-2025//Ldn/KU-2075 population, as described in our previous report [22]. The genotyped markers were assigned to the genetic map around *Net2* using the MAPMAKER/EXP version 3.0 package (Cambridge, MA, USA) [53].

## Figures and Tables

**Table 1:** Summary of RNA sequencing results for four pairs of bulks of synthetic hexaploid wheat and for tetraploid wheat cv. Langdon.

Samples	Total Read Pairs	Filtered Read Pairs (%) ^a^	Aligned to the *Ae. tauschii* Transcripts ^b^ (%) ^c^	
			KU-2075	KU-2025
Synthetic hexaploids				
non-carrier-SP1-1st	4,202,114	2,799,202 (66.61%)	2,020,405 (72.18%)	1,930,400.5 (68.96%)
non-carrier-SP1-2nd	4,059,840	2,858,956 (70.42%)	2,062,291 (72.13%)	1,971,110.5 (68.95%)
non-carrier-SP2-1st	4,492,358	2,953,088 (65.74%)	2,164,656 (73.3%)	2,083,120 (70.54%)
non-carrier-SP2-2nd	4,115,352	2,864,271 (69.60%)	2,098,383 (73.26%)	2,020,752.5 (70.55%)
*Net2*-SP1-1st	4,710,499	3,148,652 (66.84%)	2,208,392 (70.14%)	2,110,452.5 (67.03%)
*Net2*-SP1-2nd	4,403,630	3,108,568 (70.59%)	2,178,403 (70.08%)	2,082,004 (66.98%)
*Net2*-SP2-1st	4,828,182	3,249,596 (67.30%)	2,420,056 (74.47%)	2,348,814 (72.28%)
*Net2*-SP2-2nd	5,216,082	3,709,478 (71.12%)	2,763,471 (74.5%)	2,684,019 (72.36%)
Tetraploid wheat				
cv. Langdon	6,316,174	4,372,660 (69.23%)	2,974,277 (68.02%)	2,661,487 (60.87%)

^a^ The ratio of the filtered read pairs to the total read pairs. ^b^ Nishijima et al. [27]. ^c^ The ratio of the aligned reads to the filtered read pairs.

**Table 2:** The number of single nucleotide polymorphisms (SNPs) detected in four bulks of synthetic hexaploid wheat and tetraploid wheat cv. Langdon compared to the two parental *Ae. tauschii* transcriptomes.

Transcripts ^a^	KU-2075		KU-2025	
The Number of SNP	Total	Anchored to the Genome ^b^ (%)	Total	Anchored to the Genome ^b^ (%)
Synthetic hexaploids				
non-carrier-SP1	277,605	275,799 (99.35%)	262,966	261,128 (99.30%)
non-carrier-SP2	276,564	274,772 (99.35%)	269,175	267,249 (99.28%)
*Net2*-SP1	318,046	315,859 (99.31%)	296,819	294,739 (99.30%)
*Net2*-SP2	298,496	296,419 (99.30%)	285,798	283,684 (99.26%)
Tetraploid wheat				
cv. Langdon	429,346	421,957 (98.28%)	350,871	345,657 (98.51%)

^a^ Nishijima et al. [27]. ^b^ Luo et al. [39].

**Table 3:** The number of SNPs classified into three categories, including D-genome-specific allelic variations, homoeologous polymorphisms, and unclassified (those falling into neither of the other two classes).

Chr.	D-genome-Specific	Homoeologous	Unclassified	Total
1D	1674	29,307	11,760	42,741
2D	2295	33,822	13,975	50,092
3D	1611	31,932	15,532	49,075
4D	1698	28,781	11,776	42,255
5D	2936	34,961	14,336	52,233
6D	3730	24,534	10,966	39,230
7D	3983	28,593	11,606	44,182
Total	17,927	211,930	89,951	319,808

**Table 4:** List of the derived cleaved amplified polymorphic sequence (dCAPS) markers developed in this study.

Marker Name	Primer Sequence (5′ to 3′)	Restriction Enzyme
*bsa1*	TCATGACCTGCTGGTTTGTT	*Sty*I
	GATTCCAATGTTATTTCTGAACCCT	
*bsa2*	TCACAACATTCGCAGGTCAT	*Hpa*II
	TGGTTCTGTTGATCTCACTGCC	
*bsa4*	ACAAGTCGGATATCGCCAAA	*Hin*fI
	CAGCTAAAAACTGTTTGCTTGAGA	

## Abbreviations

RNA-seq	RNA sequencing
NGS	next-generation sequencing
BSA	bulked segregant analysis
SNP	single nucleotide polymorphism
Indel	insertion/deletion
NR	non-redundant
SP	Segregating Population
dCAPS	derived cleaved amplified polymorphic sequence
Ldn	Langdon
